# *Vitabel*: A Python Framework for Visualizing and Labelling High-Resolution Physiological Data for Critical Care Machine Learning

**DOI:** 10.1007/s10916-026-02417-x

**Published:** 2026-06-09

**Authors:** Simon Orlob, Wolfgang J. Kern, Benjamin Hackl, Jan Wnent, Jan-Thorsten Gräsner, Martin Holler

**Affiliations:** 1 Institute for Emergency Medicine, University Hospital Schleswig-Holstein, Arnold-Heller-Straße 3, 24105 Kiel, Germany; 2 Department of Anesthesia and Intensive Care Medicine, Medical University of Innsbruck, Anichstraße 35, Innsbruck, 6020 Austria; 3 Medical University of Graz, Neue Stiftingtalstraße 6, Graz, 8010 Austria; 4 Department of Mathematics and Scientific Computing, University of Graz, Heinrichstrasse 36, Graz, 8010 Austria; 5 IDea_Lab - The Interdisciplinary Digital Lab at the University of Graz, University of Graz, Leechgasse 34, Graz, 8010 Austria; 6 BioTech-Med Graz, Mozartgasse 12/II, Graz, 8010 Austria; 7 Department of Anaesthesiology and Intensive Care Medicine, University Hospital Schleswig-Holstein, Arnold-Heller-Straße 3, 24105 Kiel, Germany

**Keywords:** Critical care, Resuscitation, Python, Software, Labelling, Time series data

## Abstract

Artificial intelligence offers great opportunities in critical care, particularly when a vast amount of continuously acquired physiological data is incorporated. High-quality, reliably labelled data are paramount for developing and training artificial intelligence methods. However, routinely recorded data in critical care are often noisy, and the sheer volume of high-resolution data is challenging to manage. Generalizable solutions for these problems are lacking, restricting progress. To address these barriers, we developed *Vitabel*, an open-source *Python* framework for post hoc loading, visualizing, aligning, and annotating medical time series. The framework provides sensible defaults and interactive components for efficient use in preconfigured workflows, while remaining flexible and extendable for custom analysis and annotation pipelines. It integrates seamlessly into *Jupyter Notebooks*, providing an interactive, customizable interface for visual interaction with the data. In this publication, we demonstrate its utility across three use cases. The code and exemplary data are provided as browser-based demos. *Vitabel* is freely available and published under the MIT license accompanying this publication.

## Introduction

Timely, exceptional, complex decision-making characterizes critical care medicine. The heterogeneity of patients, their dynamic physiologic state, and interactions of concomitantly applied therapies challenge classic investigative approaches [1]. Simultaneously, vast amounts of data are generated by monitors, life support systems, and various diagnostic tools, making critical care a promising field for data-driven investigations and the application of artificial intelligence to gain knowledge and improve decision-making, especially when also incorporating waveform data [2]. However, any data-driven approach relies on comprehensive, clean, and reliably labelled data as ground truth [3]. This requires aggregating and harmonizing various data sources, including data alignment.

Although data capturing from medical devices, even as full-resolution waveforms, was democratized by solutions like *VitalDB* and *VSCapture* [4, 5], data access remains challenging due to proprietary data formats and varying data definitions. Furthermore, most real-world data in critical care are inherently noisy, requiring manual review and data cleansing to ensure reliability. Ultimately, to make these data suitable for data-driven methodologies and artificial intelligence, high-quality labels must be obtained, necessitating manual screening and annotation.

Several tools already support parts of the analysis of physiological time series. For example, the *WFDB* ecosystem and PhysioNet’s *LightWAVE* are well established for storing, accessing, and inspecting waveform data, particularly in the context of PhysioNet [6, 7]. Desktop tools such as *LabChart* and *EDFbrowser* provide practical visualization environments [8, 9], while *PyBioS* and related software focus on cardiovascular signal processing and variability analysis [10]. However, these tools generally emphasize specific formats, repositories, or analysis domains. In critical care research, there remains a need for a flexible and extendable framework that combines heterogeneous data loading, visual alignment of timelines, interactive manual annotation and revision, automatic label generation, and export of curated data and labels within the scientific *Python* ecosystem.

**Fig. 1:**
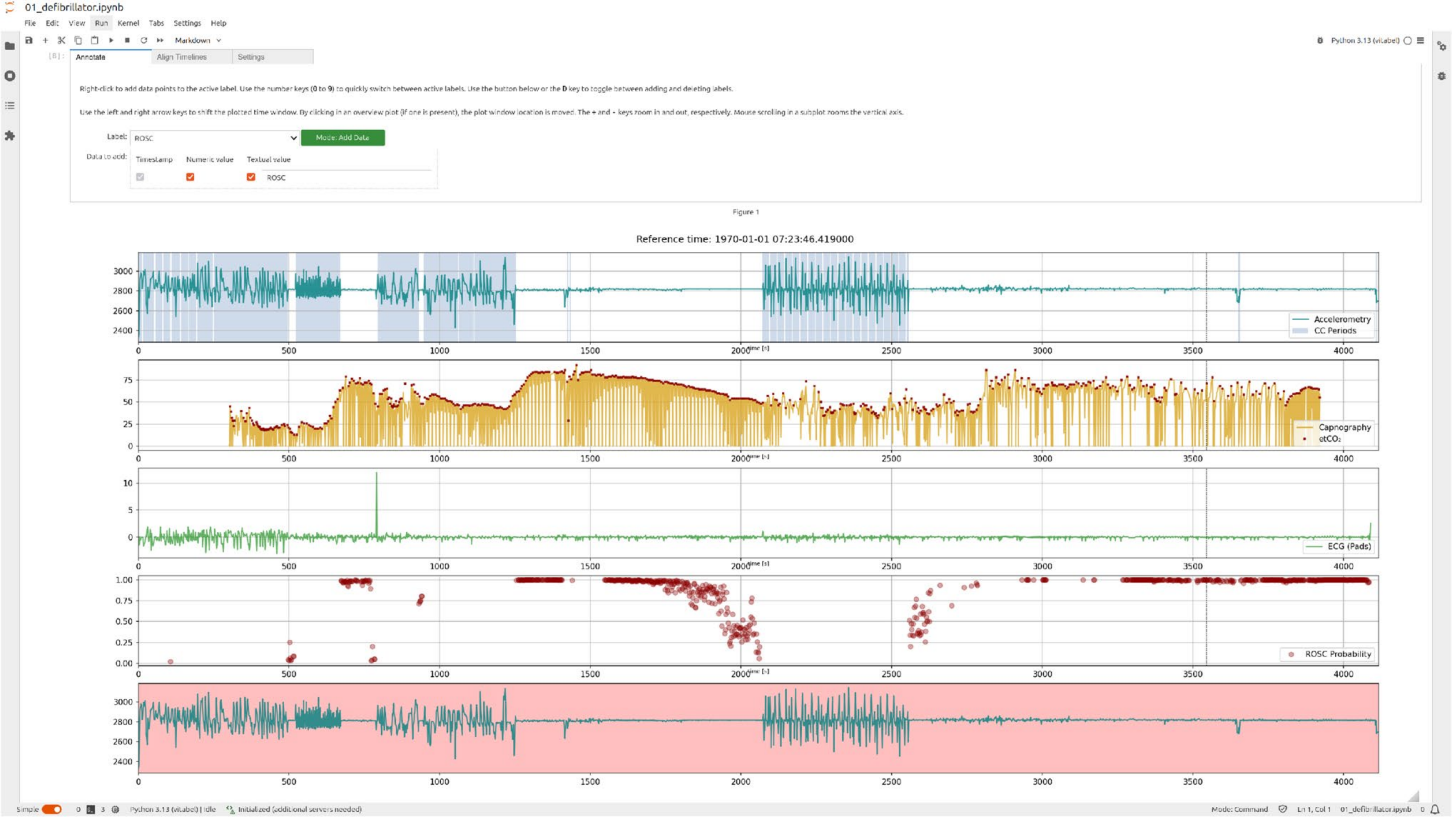
Screenshot from a *Vitabel* interactive plot. The first four subplots show detailed views of the data. From top to bottom: 1^st^ subplot: accelerometer of a CPR feedback device (teal line), chest compression periods (light blue areas); 2^nd^ subplot: capnogram (yellow line), end-tidal CO$_{2}$ (small dark-red rectangles); 3^rd^ subplot: electrocardiogram (green line); 4^th^ subplot: predicted probability of spontaneous circulation (red dots). The bottom subplot is an overview of the total accelerometer recording. The red window indicates the current position of the detailed subplots above (in this case the entire recording duration). The tabs on top allow switching between ‘Annotation’, ‘Align Timelines’, and ‘Settings’. The *Jupyter Notebook* to generate this view is available following the link in Table 1

We hereby present *Vitabel* (a portmanteau of vitals and label) as a free, open-source *Python* software framework to:

1. load time series data from various sources, ranging from high-resolution waveform data to single event time points
2. visualize them in intuitive, interactive plots that are readily interpretable by domain experts (see Fig. 1)
3. visually align different data sources in time
4. automatically preprocess data and add derived labels
5. manually customize labels and visually annotate the data with them
6. store the original data, applied manipulations, and aggregated results in standardized open data formats to ensure accessibility, interoperability and reusability

The framework is designed for post hoc analysis of recorded data in a research context, rather than for real-time processing at the patient bedside. It is intended to be directly usable by domain experts through sensible default behavior, while also enabling technically experienced users to build notebook-centric interactive interfaces tailored to specific use cases. This same design keeps the framework flexible and extensible for custom workflows. The primary interaction model of *Vitabel* is based on *Jupyter Notebooks*, keeping code, visual interaction, and documentation of processing steps in a single environment that is well established in data-driven research and computational science.

**Fig. 2:**
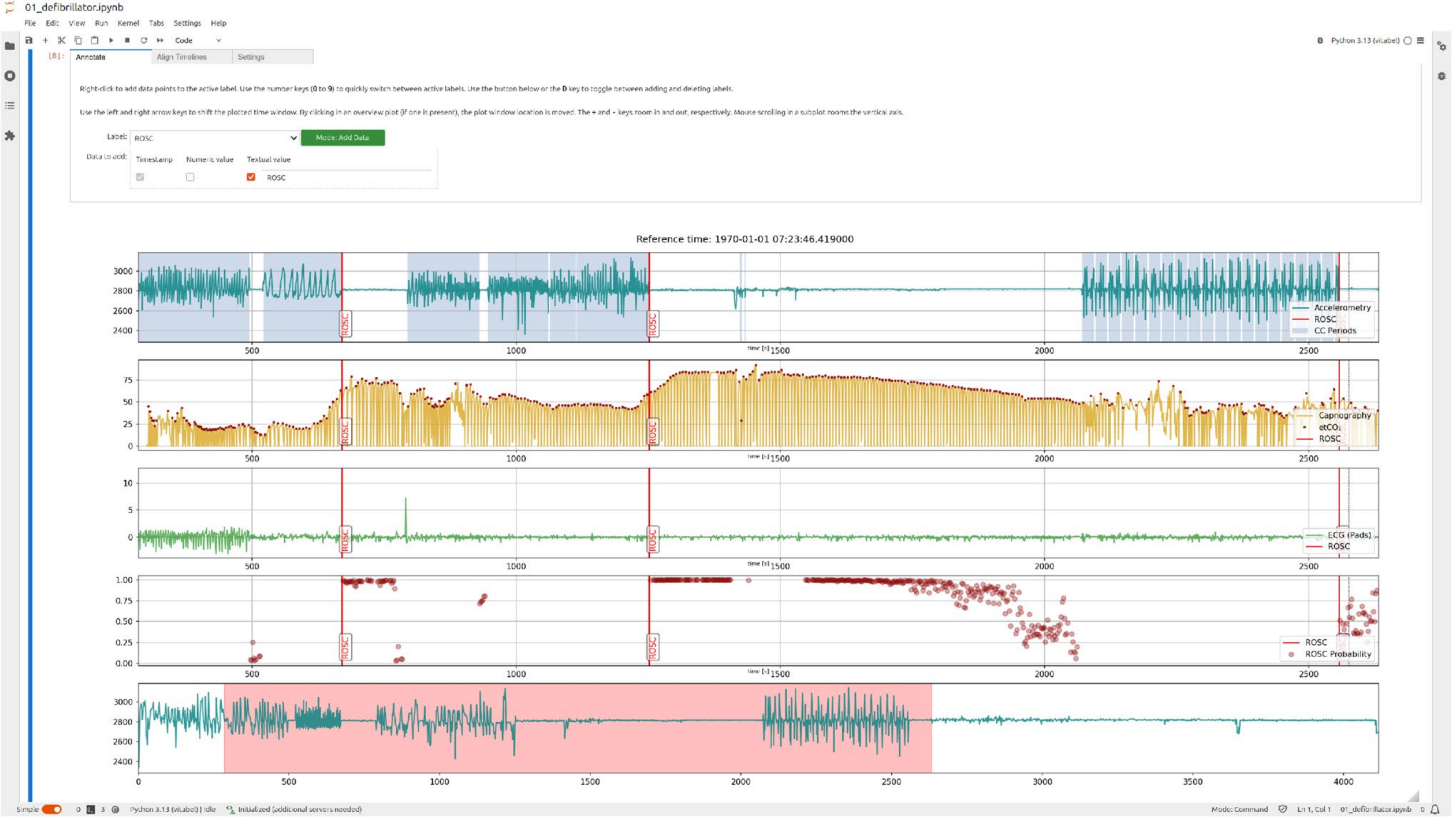
Screenshot from Use Case 1 showing the interactive plot for annotation. The first four subplots show detailed views of the data, and the bottom subplot is an overview of the entire recording (detailed description is given with Fig. 1). Vertical lines and additional text indicate the manually annotated returns of spontaneous circulation. In the annotations mode shown in this screenshot, the label to be edited can be chosen from the drop-down list. The type of data to be added to the entry can be defined by the checkboxes. By clicking the green button or pressing ‘d’ the mode can be changed from ‘Add Data’ to ‘Delete Data’ and vice versa. A detailed, interactive version of the plot is available online [20]

## Methods

### Data Model

The main container to process and store data in the proposed *Python* package named *Vitabel* is a Vitals object. A Vitals object is a collection of *Vitabel*’s two fundamental data classes: channels and labels. Both data classes represent time series data, where time can be absolute or relative. A time series is an array of time points, supporting arbitrary spacing between single instances. This plain time-series representation is also the minimal standard required for vendor-independent data: data have to be expressible as one time index, either absolute timestamps, relative time deltas, or numeric time values with a specified unit, and optionally one corresponding vector of values. For tabular data, the *pandas DataFrame* index must conform to this standard; CSV files can be configured through standard *pandas* read options. It is important to note that a time series is not required to contain associated values, enabling the representation of time-only occurrences, such as the time of specific events like a chest compression during cardiopulmonary resuscitation. Despite their shared structural characteristics, even allowing for potential interchangeability, the role and purpose of these two classes are distinctly different. Detailed explanations of both classes are provided in Sections “Channels” and Labels”, respectively. Additional information, like demographics and diagnoses, which are not time series, can be stored as metadata of a Vitals object.

#### Channels

The channels of a Vitals object contain all the time series data that are not intended to be changed after initialization. Thus, they comprise predominantly biomedical signals recorded by any device. Each channel is associated with a name that is not necessarily unique and may also contain additional, extendable, detailed metadata, including the channel’s origin and the specific recording device used. The only modification allowed for channels inside the interactive alignment workflow is a shift of the entire channel in time to manually align data due to the potential presence of unsynchronized clocks in different recording devices, while the recorded signal values remain unchanged. For shifts, the applied offset is stored separately from the original data and applied automatically when the data is queried. In addition, programmatic linear time-axis rescaling is available for recordings with temporal drift; this creates a rescaled time-series representation while preserving the original signal values. Additional information on the channel can be stored as metadata in the channel itself.

#### Labels

The labels of a Vitals object contain data either derived from channels or added upon them. Labels are subsequently modifiable. Similar to channels, labels also represent time series data. The content of label values is diverse: entries can be empty for time-points (e.g. induction of anesthesia), numerical values for regular time series (e.g., end-tidal CO$_2$ value of expiration), or textual strings for event annotations (e.g., name of an administered drug). Labels are implemented such that numerical and textual information can simultaneously be associated with a given data point. This enables, for example, the labelling of a bolus administration of a medication with a numerical dose and the corresponding drug name as text.

Interval labels are a special type of label, in which every entry is defined by a start and an end point to mark specific periods (e.g. interval of hypotension or sampling of an arterial blood gas with temporal obstruction of blood pressure measurement).

Labels can be global or local; local labels are attached to a single channel. Global labels contain information relevant to the entire recording. They are derived from assessing multiple channels of different origins, such as the time of return of spontaneous circulation in a cardiac arrest case. On the other hand, local labels are linked to a single channel, intended to represent specific points or intervals in the time series of that channel (e.g., end-tidal CO$_2$ values in a capnography channel or invalid signal intervals of a specific channel). As such, changes to the temporal domain, like an alignment of a channel, will also apply to the local (attached) label.

Labels can also contain metadata. For example, in the case of a consensual annotation, where two investigators label the same data point, metadata can be used to store information on the label source and the annotation process to differentiate the labels of the same instance. Based on their metadata, labels and channels can be retrieved.

In contrast to channels, labels can be created and modified by both automatic routines and manual annotation. It is possible to add or remove individual data points. Additionally, *Vitabel* already provides methods for automated label computation, primarily focused on data related to cardiac arrest and cardiopulmonary resuscitation, but also others like the quantification of hypotensive episode metrics [11].

### Workflow & Functionality

The typical workflow when working with a Vitals object involves four steps:

1. Loading Data
2. Processing Data, including Automatic Labelling
3. Interacting with Data
  1. Aligning timelines
  2. Labelling data
4. Storing Data

#### Loading Data

After initialization of a Vitals object, it is necessary to load the data. *Vitabel* supports loading data from generic vendor-independent time-series tables and a variety of medical devices. Generic data import into *Vitabel* comprises methods to load plain time-series data stored in *Python dictionaries*, *pandas DataFrames* [12], or CSV files. Currently, implemented device-specific importers include data from various patient monitors, anesthesia machines and syringe pumps recorded by *VitalDB* as *.vit files [4]; EDF+ files; defibrillator data from ZOLL (ZOLL Medical Corporation, Chelmsford, Massachusetts, United States) exported as JSON, XML, or paired TXT/XML files; Stryker/Physio-Control LIFEPAK data exported as XML from CodeStat software (Stryker, Kalamazoo, Michigan, United States); Corpuls data exported in *BioSemi Data Format* (BDF) (corpuls — GS Elektromedizinische Geräte G. Stemple GmbH, Kaufering, Germany); data from the *LUCAS* mechanical CPR device exported as XML files (Stryker, Kalamazoo, Michigan, United States) and EOlife ventilatory feedback (Archeon Medical, Besançon, France) exported as CSV. A detailed overview of supported importers and preconfigured channel names is provided in Table 2 (Appendix A). The framework is therefore not restricted to predefined vendors or vital-sign names. Arbitrary channels can be loaded and plotted, while common clinical signals and measurements are displayed with sensible predefined plot styles when their names match the defaults. A Vitals object can contain an arbitrary number of time series with different sample times and can thus be used to collect and store this variety in a standardized format in a single file. By collecting time series data from various files, *Vitabel* facilitates data harmonization. Because each case must fit in working memory, ingesting terabytes of raw intensive-care data into a single Vitals object is not practical. A scalable strategy is to process data case-wise, device-wise, or in other bounded chunks and subsequently aggregate derived labels and results. By doing so, the workflow decomposes a big-data problem into numerous small, manageable analyses.

#### Processing Data

*Vitabel* offers several methods to process and modify data. The available methods primarily prepare the data for further use within the interactive plot. This includes:

- Renaming channels
- Initializing empty labels for subsequent annotation in the interactive plot
- Removing unnecessary channels from the Vitals object
- Adapting plot styles of channels and labels for interactive plotting

Furthermore, a core feature of *Vitabel* is the automatic processing and annotation of certain vital signals. Concrete examples include the automatic detection of periods of chest compression [13, 14], the prediction of spontaneous circulation on accelerometer sensors from CPR feedback devices and ECG [15], and the detection of ventilations and computation of end-tidal CO$_2$ values based on capnography by Aramendi et al. [16], a method to derive respiratory phases in intra-arrest ventilation from flow and airway pressure [17] to subsequently calculate corresponding volumes [18], along with a method to calculate the area under the curve of specific blood pressure thresholds [11]. These methods either return global values, add derived channels (e.g. resampled measurements), or automatically generated labels.

#### Interacting with Data

##### Basic Controls

The key feature of *Vitabel* is its interactive plotting routine with integrated shifting and labelling functionality. The interactive plotting is based on the well-known and widely used *matplotlib* package [19]. A plot can be initialized by specifying the channels and labels to display. Besides detailed plots, the chart can also include overview plots at the bottom to indicate the current position of the detailed plot. This interface resembles a front-end commonly seen in video editing software, where the entire recording duration is presented in an overview, with a detailed view of a specific subsection displayed above (see Fig. 1).

The plot style of a channel or label can be adapted by changing its plotstyle attribute. Predefined plot styles are already provided as sensible defaults for commonly loaded channels from patient monitors like electrocardiograms, arterial blood pressure waveforms, or capnography. The color codes align with the familiar standards clinicians are used to in daily practice, ensuring intuitive recognition and seamless integration into their workflow. After initialization, the plot can be customized further by accessing the underlying *matplotlib* figure.

The interactive plot features include stepping forward and backwards in time via the arrow keys on the keyboard, zooming in and out along the time axis via the ‘+’ and ‘-’ keys, and jumping to another point in time by clicking on the corresponding position in the overview plot. The amplitude of the signal can be scaled by scrolling with the mouse wheel over the respective subplot. Alternatively, the vertical plot limits can be adapted in the ‘Settings’ tab of the plot.

Visual data interaction in the plot currently comprises functionalities such as aligning timelines and labelling data. Users can change between functionalities by switching between different tabs of the displayed plotting widget. Beyond these predefined interaction modes, the plotting components can also be embedded into custom notebook-based interfaces tailored to specific research workflows, as demonstrated in Use Case 3.

##### Aligning Timelines

*Vitabel* offers methods to shift channels and labels in time. This is useful when the clocks of different devices during recording have not been synchronized. The user can choose the channels and labels to shift by a multi-selection list. The first right mouse click sets the start point of the shift, and a red line indicates this point in time. The second right click sets the target point of the shift. The time series is updated and the plot is adapted immediately. Ultimately, by applying the offset to all channels out of sync, a cleaner and more consistent dataset is created. When signals have different native sampling rates, no resampling is required for visualization or storage, because each channel retains its own time index. The interactive plot currently supports manual constant-offset correction. If a recording shows temporal drift rather than a constant offset, a linear rescaling of the time axis can be applied programmatically; this supports cases in which the relative shift changes approximately linearly over time. While local labels tied to a specific channel are automatically shifted with their channel during offset correction, global labels need to be shifted explicitly. For pure shifts, the relative time delta for the individual signal is stored as an ‘offset’ attribute, while the original timestamps are maintained to preserve reproducibility. Linear rescaling can be reproduced from the corresponding programmatic transformation parameters.

##### Labelling Data

Previously auto-generated or manually added labels that are currently displayed can be selected from the drop-down list within the annotation menu in order to remove, add or alter data points. The labels can also be chosen rapidly with keyboard number keys, depending on their order in the list. The data type of the label entry can be specified using the checkboxes below; entries can contain no data, numerical data, textual data, or both. An entry to the label is added by right-clicking at the desired position in the subplot. For text labels, text can be entered into a text field before clicking. In the case of numerical data, the position of the mouse pointer is translated into the corresponding *y*-value of the subplot. To delete an annotation, the user needs to toggle delete mode by clicking the button or pressing the ‘d’ key. The plot is updated each time an entry is added to or deleted from a label. The labels and corresponding values can then be used for further data filtering and analysis.

#### Storing Data

The entire Vitals object, including channels, labels and metadata, can be stored and reloaded later for subsequent use and modification. For filesize reduction *Vitabel* stores data in a compressed format using *bzip2*. *Vitabel* can also export individual channels and labels as CSV files for further external processing.

## Results

We demonstrate the scope and functionality of *Vitabel* through three use cases. For each, we provide code snippets and describe their application. Screenshots of the generated plots illustrate the visualization produced by *Vitabel*. The complete code and exemplary data are available in the repository as dedicated *Jupyter Notebooks*, one per use case.

These notebooks can also be explored interactively in a web browser via the links in Table 1, without requiring any local installation. This is achieved by sandboxing the notebooks in environments created using the *binder* project [12].

While the code snippets presented here are intentionally concise, the accompanying *Jupyter Notebooks* provide additional detail for researchers seeking to adapt *Vitabel* to their specific requirements.

The data model is described in Section “Data Model” and the general workflow & functionality of *Vitabel* in Section “Workflow & Functionality”.

The four main data classes that constitute the package of *Vitabel* are imported in *Python* by the following command:
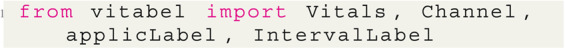

**Table 1:** Overview of the discussed use cases with links to the corresponding interactive online demo environments

Use Case	Objectives	Interactive Demo
1^st^ Out-of-Hospital Cardiac Arrest	reading the defibrillator recording, computing the probability of spontaneous circulation, visualizing them, and labelling the time-point of return of spontaneous circulation	[20]
2^nd^ Porcine Model of Cardiac Arrest	reading data from multiple devices, stored in multiple file formats, visualizing them, aligning these recordings in time, and labelling intervals of artifacts	[21]
3^rd^ Anesthesia Chart	reading trend data, medication, and surgical time-points from an anesthesia chart, computing area under the curve of mean arterial pressure below 65mmHg, visualizing the data, and adding missing anesthesiological time points, finally wrapping the plot in an user interface	[22]

**Fig. 3:**
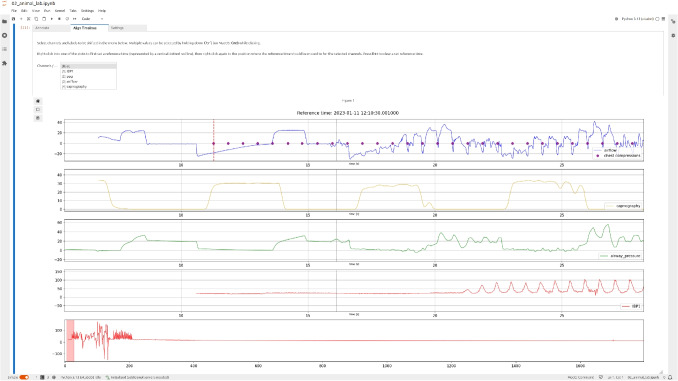
Screenshot from the alignment process in Use Case 2. The layout of the figure is similar to Fig. 2. The first four subplots show detailed views of the data (1^st^ subplot: blue line - airflow, purple dots - single chest compressions; 2^nd^ subplot: yellow line - capnogram; 3^rd^ subplot: green line - airway pressure; 4^th^ subplot: red line - invasive arterial blood pressure). The bottom subplot is an overview of the total invasive arterial blood pressure recording. The channels that should be shifted can be selected from the multi-select list. By clicking with the right mouse button, the starting point of the shift was defined and marked by a vertical, dashed red line. Subsequently, the target point can be selected by clicking again with the right mouse button on the plot (e.g. artifact of the 1^st^ chest compression in the airflow signal). A detailed, interactive version of the plot is available online [21]

**Fig. 4:**
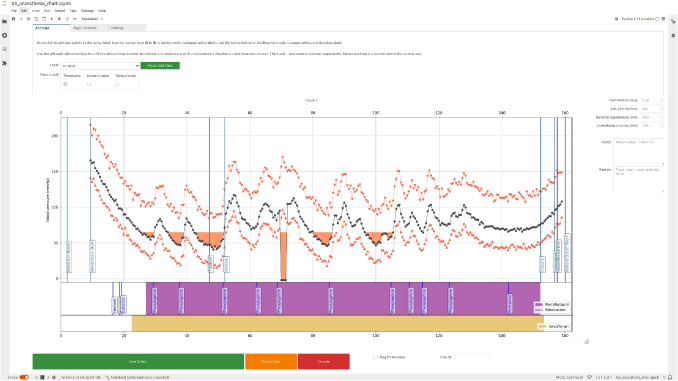
Screenshot from the third use case with data from an artificial anesthesia chart. The 1^st^ subplot displays the blood pressure (systolic, mean, and diastolic arterial pressure) and labels for relevant surgical time points, extracted from the patient data management system. In this figure no overview plot is provided and the lower two subplots contain only labels to display medications (bolus and continuous), which also have been automatically generated by data extraction from the patient data management system. The plot is integrated into a user interface with buttons, text inputs and fields with metrics to review. The red vertical line indicates the manually placed time point for the ’Analysis’ label, which defines the segment used for statistical analysis in this example. A detailed, interactive version of the plot is available online [22]

### First Use Case: Emergency Medical Service

Use case 1 shows the handling of a single defibrillator recording of a real-world out-of-hospital cardiac arrest (see Fig. 2). The objective of this use case is to review the resuscitation attempt and manually label the occurrence of return of spontaneous circulation.

In the first step, the file extracted from the defibrillator is loaded.
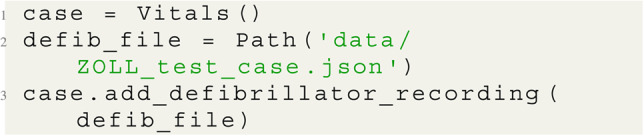


Afterward, new labels for single ventilations and end-tidal CO$_2$, derived from capnography, as well as the probability of spontaneous circulation, derived from accelerometer and electrocardiogram, are automatically created, with the methods implemented in the package [13, 15, 16]. Detailed documentation of the functions can be found in the package's *Read the Docs*.
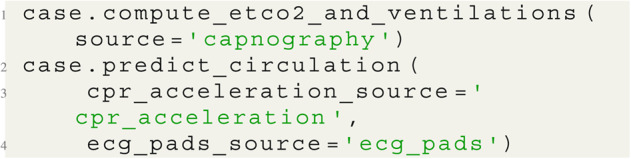


In preparation for the subsequent labelling in the interactive plot, a new global label for the time of return of spontaneous circulation is created, including metadata and plot style definitions.
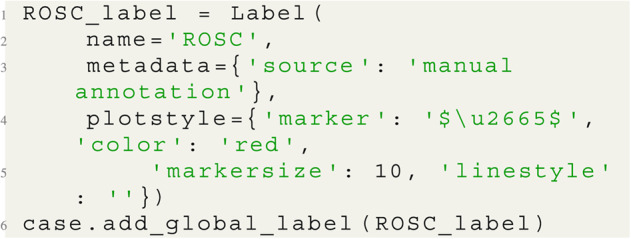


The subplots are defined by lists within a list ([[],[]]). Each inner list defines the channels/labels of the specific subplot. The number of lists defines the number of subplots. If a subplot should not contain any channel or label, an empty list has to be given ([]). Overview subplots are defined in the same way and are appended underneath. In this case, the automatically generated labels (’cc_periods’, ’etco2_from_capnography’, ’rosc_probability’) are displayed on top of channels. The labels with the time of return of spontaneous circulation will be depicted in all subplots; occurrences can be added manually. A screenshot of the interactive plot is also shown in Fig. 2.
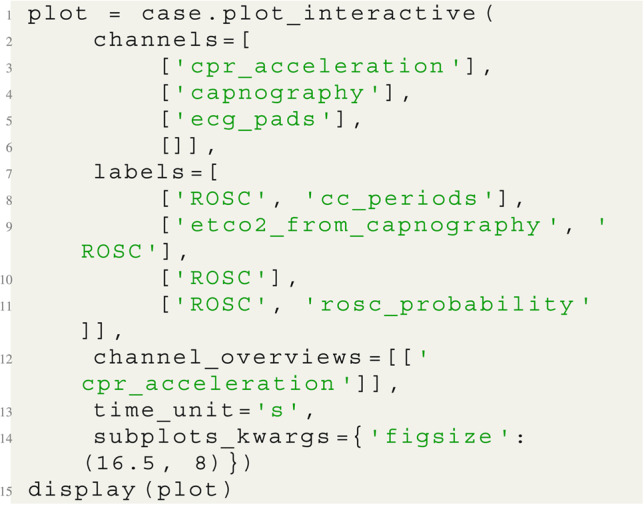


The data is stored after annotation. Additionally, the ’ROSC’ label is saved in a CSV file.
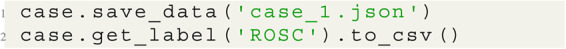


### Second Use Case: Animal Laboratory

The second exemplary application concerns an animal laboratory experiment on ventilation during cardiopulmonary resuscitation, depicted in Fig. 3. Various measurements were collected during this experiment using different devices, including chest compression events of Stryker’s *LUCAS*, patient monitor data captured by *VitalRecorder* [4], and ventilatory monitoring. The objective of this use case is to combine recordings of different devices and align their data.

In this example, three different input formats are loaded: the *LUCAS* XML export containing chest compression events, the *VitalDB* *.vit file containing patient-monitor data such as capnography and invasive blood pressure, and a compressed CSV file containing the ventilatory flow signal. These data are imported by calling the following functions:
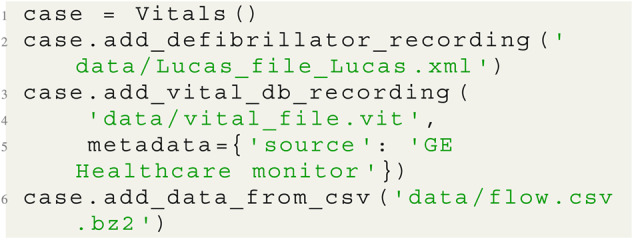


An interval label is initialized and attached to its respective channel. In the code below, aline_noise is the name of a manually created IntervalLabel used to mark noisy periods in the invasive arterial blood pressure channel; it is not a built-in noise-detection function. Before plotting, plot styles for channels and labels are set to adapt the appearance of the plot.
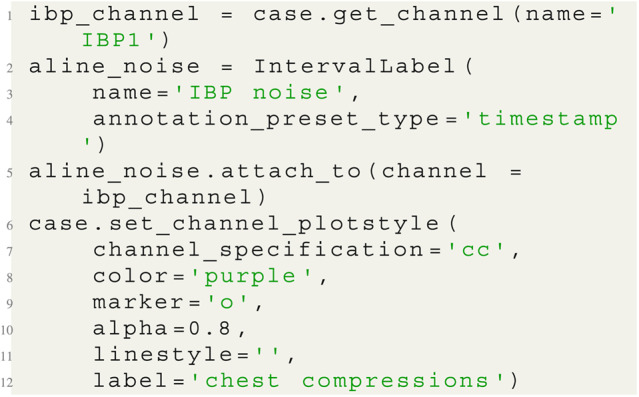


Next, we generate an interactive plot with the channels and labels we want to work on.
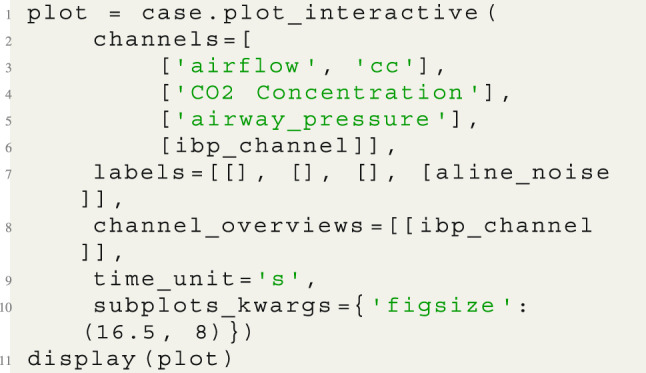


The three data sources (ventilatory monitoring, chest compressions of *LUCAS* and the patient monitor) had unsynchronized clocks. This can be seen in the artifacts and pulse waves generated by chest compressions. Thus, the compression markers should be shifted by aligning the first marker with the first occurrence of reversed airflow caused by chest compression [18]. Furthermore, the offsets between blood pressure and the remaining signals can be corrected. If a constant offset is insufficient, for example because the device clocks drift over the course of a long recording, a linear time-axis rescaling can be applied programmatically outside the interactive plot.

These alignments can be performed in the ‘Adjust Timelines’ tab in the header of the interactive plot. This functionality is also depicted in Fig. 3. After the alignment, all signals are prepared for further analysis.

Artifacts in the blood pressure signal can be labelled manually with the aline_noise interval label. Thereby, these segments can be excluded from further analysis by filtering the channel using the labelled interval label. The necessary menu to manually label can again be activated by the ‘Annotate’ tab. For an interval label, two separate clicks are required to define the beginning and end of the interval.

All data and labels are stored afterward.
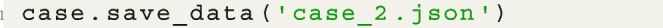


### Third Use Case: Operating Theatre

The third example considers anesthesia charts from surgery. Due to data privacy regulations, artificial data imitating an anasthesia chart are used instead of real-world data. The objective of this example is to quantify intraoperative hypotension. To achieve this, the period for the analysis has to be defined, and erroneous readings have to be excluded.

The data were previously loaded into *Vitabel*, stored, and are reloaded for this example. A new label ‘Analysis’ is created to label the edges of the analysis interval and added to the Vitals object. In contrast to the previous examples, this example does not contain continuous waveform data but discrete blood pressure values and labels for administered medications.
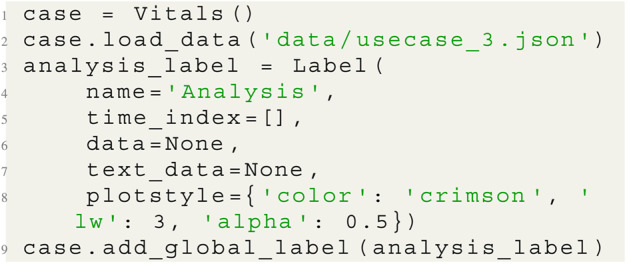


To correct erroneous measurements of the mean arterial pressure, a label ’MAP’ with the values from the channel ’MAP’ is generated. As labels have the property to add and delete entries, this allows for visual exclusion of data points.
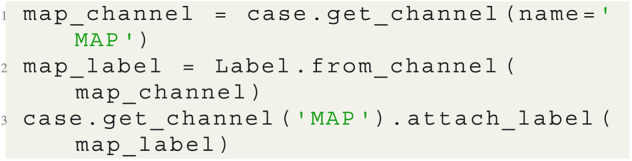


Afterward, an interactive plot is created, but not yet displayed. Instead, the underlying figure and axes are accessed to further customize the plot.
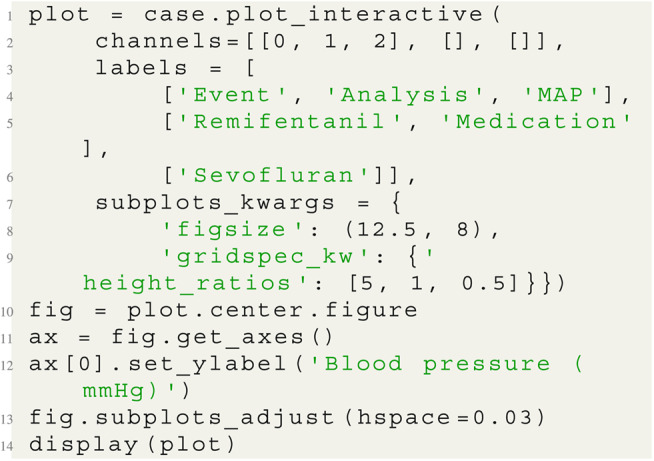


The new label ’Analysis’ can be used to label the induction of and emergence from anesthesia. The interactive plot, embedded in a graphical user interface, is shown in Fig. 4. This interface allows the user to iterate over a series of cases, highlighting hypotensive episodes and displaying hypotension metrics. This use case illustrates that *Vitabel* can serve not only as a generic annotation interface, but also as a building block for task-specific notebook-based user interfaces around the same underlying data structures and plotting functionality. The code to implement this is explained in detail in the specific *Jupyter Notebook* (see Table 1).

## Discussion

The main contribution of *Vitabel* is a framework for post hoc curation, alignment, annotation, and analysis of physiological time series data in critical care research. In this setting, the main bottleneck is often not the downstream statistical or machine-learning method itself, but the preparation of heterogeneous, noisy, and incompletely labelled recordings into a suitable form. As such, *Vitabel* addresses a fundamental step in any data-driven research project: consolidation of reliable ground truth.

The three use cases illustrate complementary aspects of this workflow. The first use case shows how automatically derived labels and manual review can be combined during the annotation of defibrillator recordings. The second use case demonstrates the alignment of data from multiple unsynchronized devices and the annotation of artifacts in a heterogeneous multimodal recording. The third use case shows that *Vitabel* is not limited to a generic plotting interface, but can also serve as a building block for task-specific notebook-based user interfaces built on the same underlying data structures and plotting functionality. Collectively, these examples show that *Vitabel* is intended as a flexible framework for research workflows rather than as a fixed-purpose viewer.

This integrated workflow is particularly relevant in critical care research, where recordings frequently originate from multiple devices with unsynchronized clocks and differing data formats, and where reliable ground truth often depends on manual review by domain experts. By enabling these tasks within a common environment providing immediate visual feedback, *Vitabel* may reduce the practical burden of data curation and support the creation of cleaner and more reproducible datasets for downstream analysis.

For domain experts, *Vitabel* offers access to data with high sample rates even if they have little experience in working with the complexity behind these data. Sensible defaults, *Jupyter Notebooks* prepared by data scientists, and browser-based demo environments can lower the barrier for domain experts to work with extensive time series data. Of note, with tools like *Binder* and *Voilà,* applications of *Vitabel* can be published immediately, as our web-based, interactive visualization tool for raw data inspection demonstrates [17]. This can ultimately increase transparency of the research process.

For users with more technical background the framework remains extensible. Custom loaders, preprocessing routines, and task-specific interfaces can be implemented. In this sense, *Vitabel* is not primarily a stand-alone end-user tool, but a scientific *Python* framework designed to support interdisciplinary collaboration between clinicians, researchers, and developers.

Furthermore, *JupyterLab* instances are common on high-performance computing clusters and are already established in many academic institutions. Utilizing *JupyterHub* allows for the straightforward implementation of *Jupyter Notebooks* for multiple users on a centralized server, with personalised access, enabling researchers to remotely work on and annotate centrally stored, sensitive data while complying with relevant data protection regulations.

The framework has already been applied successfully in several projects [13–15, 23]. In addition, *Vitabel* is the backbone of a recently implemented service in the German Resuscitation Registry for automated analysis of defibrillator recordings and fine-graned assessment of resuscitation quality [24–27]. The source code is published open-source under the MIT license in a repository accompanying this publication [28] which also includes the *Jupyter Notebooks* and data for the use cases discussed above (see Table 1); the latest release at the time of writing is version v0.1.1.

### Limitations and Future Directions

*Vitabel* does not provide a stand-alone graphical desktop application, and its setup and extension require basic coding literacy in *Python*. At present, the framework is centered on *Jupyter*-based workflows and an interactive plotting implementation built on top of *matplotlib*. While this design has proven effective for research workflows with post hoc annotation and collaborative notebook-based use, it may not be the most convenient entry point for all users or all deployment settings.

Future development will focus on both usability and modularization. On the usability side, this includes improved loading and storing routines, more elaborate predefined plot styles, deeper handling of standard units, easier metadata-based channel selection during timeline alignment, and convenience functions for common data-manipulation tasks.

On the architectural side, an important next step is to further separate core data structures, processing routines, and user-interface components, such that project-specific workflows and interfaces can be implemented more easily and maintained more systematically. This includes support for alternative graphical backends (e.g., *plotly* instead of *matplotlib*) and further device-specific loaders.

Beyond the core development team, the open-source model should facilitate broader community-driven development of the framework. In this way, *Vitabel* may continue to develop into a useful tool for data handling throughout the entire pipeline of data-driven research in critical care, within the scientific *Python* ecosystem.

## Data Availability

The code of the software package is available in a Zenodo repository: https://doi.org/10.5281/ZENODO.15771826. The jupyter-notebooks of the use cases are available as interactive binder containers: - https://mybinder.org/v2/gh/UniGrazMath/vitabel/v0.1.1?urlpath=%2Fdoc%2Ftree%2Fexamples%2F01_defibrillator.ipynb - https://mybinder.org/v2/gh/UniGrazMath/vitabel/v0.1.1?urlpath=%2Fdoc%2Ftree%2Fexamples%2F02_animal_lab.ipynb - https://mybinder.org/v2/gh/UniGrazMath/vitabel/v0.1.1?urlpath=%2Fdoc%2Ftree%2Fexamples%2F03_anaesthesia_chart.ipynb

## Appendix A: Supported Importers and Predefined Vital Signals

*Vitabel* can store and visualize arbitrary plain time series and is therefore not limited to the vendors or signal names listed below. The tables summarize the currently implemented device-specific importers and the vital-sign names for which default naming or plot-style conventions are provided. The actual channels available in a case depend on the source device configuration and export.

**Table 2:** Currently supported importers and expected export formats

Source	Expected export format	Supported signals and parameters
ZOLL defibrillators	JSON or XML exports; for some devices paired TXT/XML exports	ECG including pads and 12-lead channels, capnography, SpO$_2$/plethysmography, invasive and non-invasive blood pressure, CPR acceleration and compression metrics, temperature, impedance, defibrillation events
Stryker/Physio-Control LIFEPAK	CodeStat XML files including continuous, waveform, and CPR event log exports	ECG, impedance, capnography, compression and ventilation events, EtCO$_2$, SpO$_2$, non-invasive and invasive blood pressure, heart rate
Stryker LUCAS	CodeStat XML files including LUCAS and CPR event log exports	Mechanical chest-compression events and CPR-related time markers
Corpuls	BDF waveform export with accompanying event/log files	ECG, capnography, invasive and non-invasive blood pressure, impedance, CPR depth/rate/force, SpO$_2$, heart rate, respiratory rate, temperature, SpCO/SpHb/SpMet
VitalDB / VitalRecorder	*.vit files	Tracks from patient monitors, anesthesia machines, and syringe pumps, imported as channels or labels depending on track type
EDF+	EDF+ files with annotations	Biosignal channels and annotation labels
EOlife	CSV export	Ventilatory feedback data
Generic user data	*Python* dictionaries, *pandas DataFrames*, or CSV files	Arbitrary time series with a datetime, timedelta, or numeric time index and optional corresponding values
